# Is Environmental Enrichment Ready for Clinical Application in Human Post-stroke Rehabilitation?

**DOI:** 10.3389/fnbeh.2018.00135

**Published:** 2018-07-11

**Authors:** Matthew W. McDonald, Kathryn S. Hayward, Ingrid C. M. Rosbergen, Matthew S. Jeffers, Dale Corbett

**Affiliations:** ^1^Department of Cellular & Molecular Medicine, University of Ottawa, Ottawa, ON, Canada; ^2^Canadian Partnership for Stroke Recovery, Ottawa, ON, Canada; ^3^Stroke Division, Florey Institute of Neuroscience and Mental Health, Heidelberg, VIC, Australia; ^4^NHMRC Centre for Research Excellence in Stroke Rehabilitation and Brain Recovery, Heidelberg, VIC, Australia; ^5^Division of Physiotherapy, School of Health and Rehabilitation Sciences, The University of Queensland, Brisbane, QLD, Australia; ^6^Allied Health Services, Sunshine Coast Hospital and Health Service, Birtinya, QLD, Australia

**Keywords:** environmental enrichment, stroke, rehabilitation, neuroplasticity, recovery, clinical translation

## Abstract

Environmental enrichment (EE) has been widely used as a means to enhance brain plasticity mechanisms (e.g., increased dendritic branching, synaptogenesis, etc.) and improve behavioral function in both normal and brain-damaged animals. In spite of the demonstrated efficacy of EE for enhancing brain plasticity, it has largely remained a laboratory phenomenon with little translation to the clinical setting. Impediments to the implementation of enrichment as an intervention for human stroke rehabilitation and a lack of clinical translation can be attributed to a number of factors not limited to: (i) concerns that EE is actually the “normal state” for animals, whereas standard housing is a form of impoverishment; (ii) difficulty in standardizing EE conditions across clinical sites; (iii) the exact mechanisms underlying the beneficial actions of enrichment are largely correlative in nature; (iv) a lack of knowledge concerning what aspects of enrichment (e.g., exercise, socialization, cognitive stimulation) represent the critical or active ingredients for enhancing brain plasticity; and (v) the required “dose” of enrichment is unknown, since most laboratory studies employ continuous periods of enrichment, a condition that most clinicians view as impractical. In this review article, we summarize preclinical stroke recovery studies that have successfully utilized EE to promote functional recovery and highlight the potential underlying mechanisms. Subsequently, we discuss how EE is being applied in a clinical setting and address differences in preclinical and clinical EE work to date. It is argued that the best way forward is through the careful alignment of preclinical and clinical rehabilitation research. A combination of both approaches will allow research to fully address gaps in knowledge and facilitate the implementation of EE to the clinical setting.

## Early Beginnings

### History of Environmental Enrichment

Environmental enrichment (EE) was first studied by Canadian scientist Donald Hebb, who raised rats in his home and later showed they were superior to laboratory raised animals in tests of problem solving ability (Hebb, 1947). His influential book, the Organization of Behavior: A Neuropsychological Theory (Hebb, 1949), emphasized the importance of experience in shaping behavior and provided the stimulus for research examining how EE changes the brain and subsequently behavior. Much of the work in the 1960’s focused on the effects of EE on the undamaged brain. Seminal studies by Rozenzweig and others showed that brain plasticity (e.g., dendritic branching) was dramatically altered by varying experience (Rosenzweig et al., 1962; Bennett et al., 1964; Diamond et al., 1964; Greenough et al., 1973). These use-dependent neuroplastic changes can be induced across the life span and are associated with improved performance on various learning and memory tasks. Later efforts investigated how EE affected the damaged brain (Will et al., 2004). For example, studies showed that EE attenuated the effects of frontal cortex injury (Kolb and Gibb, 1991), as well as both global (Farrell et al., 2001) and focal ischemia (Ohlsson and Johansson, 1995; Johansson, 1996; Puurunen et al., 2001; Risedal et al., 2002).

Based on relatively little preclinical evidence many “so-called” neuroprotective drugs were advanced into clinical stroke trials where they met universal failure (O’Collins et al., 2006). In contrast, an overwhelming amount of preclinical evidence, accumulated over several decades, shows that EE enhances learning and memory, promotes various forms of neuroplasticity and consistently improves recovery from brain injury, including stroke. In spite of this evidence there has been limited translation of this promising intervention into the clinical setting (Livingston-Thomas et al., 2016). The purpose of this review article, is to summarize the widespread preclinical evidence for utilizing EE as a therapeutic intervention for stroke recovery and examine why EE has largely remained a laboratory phenomenon. Additionally, how preclinical and clinical investigators can facilitate the transition of EE into the clinical setting is discussed.

### Defining Environmental Enrichment

**Figure 1:**
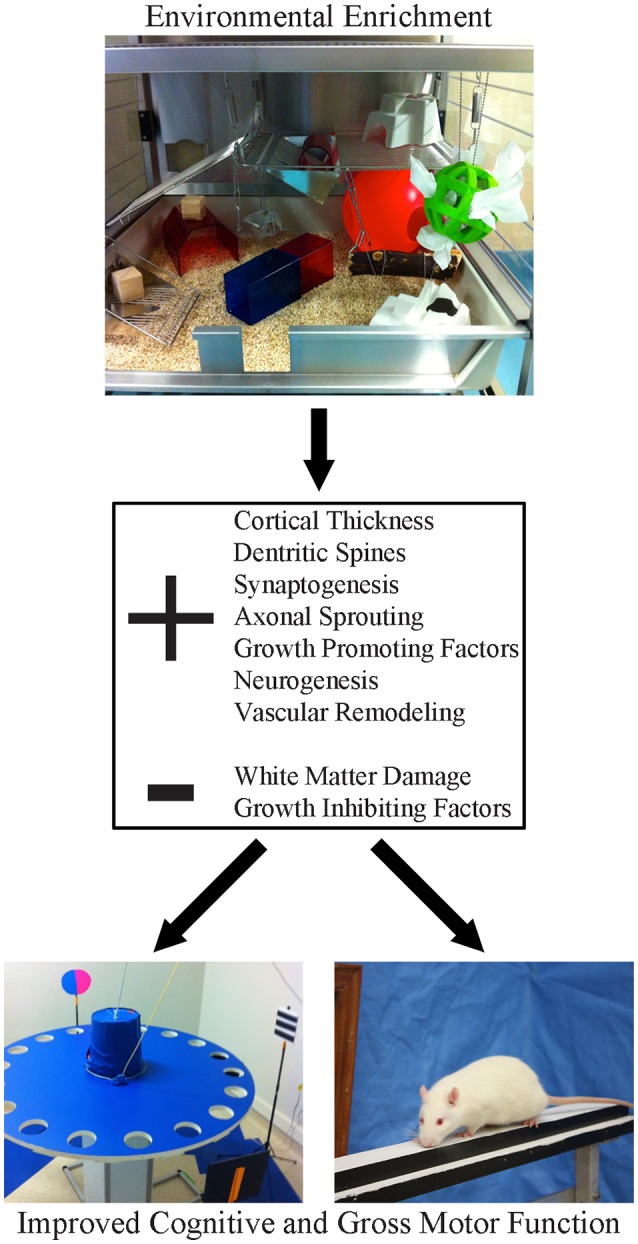
Environmental enrichment (EE) is a multi-faceted form of housing that provides enhanced motor, cognitive, sensory and social stimulation, relative to the standard conditions of rodent housing. This form of housing has been shown to create widespread changes in the neuroplastic milieu of the brain. Following stroke, these beneficial changes create a neural environment that is permissive to recovery, resulting in robust improvements in both cognitive and gross motor function.

A major impediment to clinical translation has been inconsistency in how EE is defined experimentally. This has created confusion in the clinical community because it’s unclear which EE paradigm or what critical elements of EE should be adapted for patients. As originally conceived, EE was designed to provide a more enriching, stimulating environment for animals to more closely mimic conditions encountered in the wild. There is no standardized form of EE; for some, enrichment means little more than housing several animals together in a standard sized cage containing a tube and a running wheel. Other configurations are much more elaborate and engaging, consisting of a very large, multi-level cage, that includes toys, ramps, ladders and ropes, which are replaced or moved at intervals (e.g., daily, or weekly) throughout an experiment. The elements of the enrichment cage (Figure 1) provide opportunities for social interaction, to stimulate exploration (e.g., multi-level floors connected by tubes) and engage in activities (e.g., nesting, crossing beams and hanging platforms) that tax balance, strength and provide somatosensory stimulation. The replacement of objects and changing their location within the cage provides cognitive stimulation, additional olfactory and visual stimulation and further encourages exploration and physical activity. Introduction of new materials into the cage can be used to provide added sensory stimulation (Zubedat et al., 2015). In the context of stroke recovery, it is important to recognize that EE needs to include a task specific component that targets the animals’ primary deficits. For example, upper limb impairment is very common clinically (Duncan et al., 1992; Kwakkel et al., 2003) and consequently, most preclinical investigators target the forelimb motor cortex in their stroke studies (Murphy and Corbett, 2009; Corbett et al., 2017). EE alone is not effective in promoting recovery of skilled forelimb movements (e.g., reaching; Grabowski et al., 1993), since there is no opportunity to engage in this activity in standard EE configurations. As such, to fill this void our group adds a daily reaching task component to EE which dramatically improves the level of recovery provided by EE (Biernaskie and Corbett, 2001; Biernaskie et al., 2004; Jeffers and Corbett, 2018). Thus, the ideal definition of EE, unlike typical stroke rehabilitation in the clinic, encompasses a changing environment that encourages socialization, exercise, sensory and cognitive stimulation, and task-specific therapy targeting the primary impairment.

Most animal studies provide unlimited access to enrichment 24 h a day, 7 days a week with relatively few studies using shorter enrichment exposures (Leger et al., 2015). This feature of EE raises immediate concerns with clinicians when attempting to extrapolate results from animal studies where not only the configuration of EE, but also practical concerns, limit the duration or amount of therapy time that can be allocated to EE vs. other forms of patient care. Another important consideration related to the duration of EE is that most of the demonstrated benefits in fostering stroke recovery, and the postulated mechanisms underlying these benefits, may not hold if shorter durations of EE are employed. This is an important consideration in view of translational limitations inherent in most preclinical exercise studies. For example, running wheel exercise has long been known to enhance neurogenesis (van Praag et al., 2000; Voss et al., 2013) which in turn is suggested to contribute to improvements in learning, memory, and recovery from brain injury, including stroke (Voss et al., 2013). However, access to this form of exercise, like EE, is typically provided to rodents 24 h per day. It is unclear how such prolonged exercise regimens could be possible for stroke patients who typically are older, experience fatigue, have sensorimotor impairments and are much more sedentary than age-matched controls (Bernhardt et al., 2004; Duncan et al., 2012). In animal studies, the effects on neurogenesis are much more modest when running wheel access has been limited to several hours per day on alternate days (Nguemeni et al., 2018).

A concern with the implementation of EE in the clinic is that rodents experience a relatively impoverished environment in standard animal facilities, and EE may simply normalize typical living conditions (Würbel, 2001). If this is indeed the case, then EE may not be effective in humans who are viewed as already living in an enriched, stimulating environment. However, Bernhardt et al. (2004) have shown that after stroke patients spend a large proportion of time in isolation and physically inactive (Fini et al., 2017). Further, patients frequently report the rehabilitation setting as being unstimulating and boring (Kenah et al., 2017). Thus, the early post-stroke environment for humans and impoverished animals may actually be relatively similar.

### Environmental Enrichment as a Combination Therapy

A question, often encountered when discussing the beneficial effects and potential mechanisms underlying the neuroplasticity enhancing actions of EE, is what element of the EE is most important? Is it socialization, exercise, sensorimotor activation or cognitive stimulation? There have been a number of attempts to dissect EE into the relative importance of its individual components. Prior to bilateral cortical injury, rats given 2 h per day of EE for 25 days performed better on a motor task than those given the same amount of running wheel exercise (Gentile et al., 1987). Similarly, improved motor outcomes of EE compared to running wheel exercise-alone have also been observed after middle cerebral artery occlusion (MCAo) in rats, indicating the important influence of socialization on recovery (Johansson and Ohlsson, 1996; Risedal et al., 2002). Using a modified EE paradigm in which EE was combined with daily reach training (i.e., enriched rehabilitation, ER), it was found that EE, running exercise and reach training all produce a uniform pattern of activation throughout all layers of the sensorimotor cortex after stroke, however ER causes a more specific pattern of activation, targeting layer II and layer III motor neurons (Clarke et al., 2014). Recently, we showed that ER is more effective than either EE alone or reach training alone at restoring skilled forelimb function after stroke (Jeffers and Corbett, 2018). Similarly, others have shown a synergistic benefit when EE is paired with either resistance exercise or increased social interaction (Brenes et al., 2016; Prado Lima et al., 2018).

The pattern emerging from studies using EE to promote post-stroke recovery is that the whole is greater than the sum of the parts (Jeffers and Corbett, 2018). In this regard, EE shares similarity with other pleiotropic treatments such as exercise, hypothermia and ischemic tolerance, that have proven to be effective in reducing ischemic damage to the brain (Iadecola and Anrather, 2011). Cell death, like stroke recovery, is not dependent on a single mechanism. Indeed, attempts to rescue cells from ischemic injury or restore lost function after stroke with single target interventions have been met with little success (Murphy and Corbett, 2009; Iadecola and Anrather, 2011; Corbett et al., 2014; Hayward et al., 2014; Carmichael, 2016). The advantage of using EE or ER is that these synergistic approaches engage multiple, potentially beneficial mechanisms (described below and listed in Table 1) whereas the single target approach has failed completely in stroke neuroprotection and other conditions, including Alzheimer’s disease (Iadecola and Anrather, 2011; Corbett et al., 2014). As such, EE and ER should be viewed as combination therapies that create a permissive, regenerative state in the brain that is receptive to use-dependent, task-specific forms of rehabilitation and other recovery promoting treatments.

**Table 1:** Potential underlying mechanisms of environmental enrichment (EE) beneficial for promoting stroke recovery.

EE-induced plasticity	References
↓ Lesion volume	Buchhold et al. (2007) and Zhang et al. (2017)
↑ Dendritic remodeling	Biernaskie and Corbett (2001) and Johansson and Belichenko (2002)
↑ Synaptogenesis	Jones et al. (1999), Xu et al. (2009) and Hirata et al. (2011)
↑ Axonal remodeling	Papadopoulos et al. (2009) and Li et al. (2015)
↓ White matter damage	Hase et al. (2017, 2018)
↑ Antioxidant activity	Cechetti et al. (2012)
↑ Angiogenesis	Hu et al. (2010), Matsuda et al. (2011), Zheng et al. (2011), Yang et al. (2012), Ma et al. (2013), Seo et al. (2013) and Zhang et al. (2017)
↓ BBB leakage	Hase et al. (2017) and Zhang et al. (2017)
↑ Neurogenesis	Komitova et al. (2005a,b, 2006); Buchhold et al. (2007), Wurm et al. (2007) and Venna et al. (2014)
↑ Growth-promoting factors (BDNF, Gap43, FGF-2)	Gobbo and O’Mara (2004), Ploughman et al. (2007), Mizutani et al. (2011), Seo et al. (2013) and Venna et al. (2014)
↓ Growth-inhibiting factors (aggrecan-containing perineuronal nets, NOGO-A)	Madinier et al. (2014) and Li et al. (2015)

*Up and down arrows indicate an increase or decrease in the corresponding factor in response to EE, respectively*.

## How Does Enrichment Enhance Plasticity and Recovery From Stroke?

### Underlying Mechanisms

Until the work of Mark Rosenzweig and Marian Diamond in the 1960s it was generally thought that the adult brain was fixed and unable to undergo any degree of neuroplasticity. Their work was the first to show that the brains of rats that lived in an EE weighed more, had increased cortical thickness, and demonstrated increased cortical acetylcholinesterase activity compared to their restricted littermates (Rosenzweig et al., 1962; Bennett et al., 1964; Diamond et al., 1964). In response to stroke, synaptogenesis, axonal sprouting, gliogenesis and neurogenesis are significantly upregulated, creating an environment that is highly permissive to behavior-driven plasticity (Murphy and Corbett, 2009; Zeiler and Krakauer, 2013; Carmichael, 2016). It is now recognized that an EE stimulates a number of neuroplastic processes, such as structural changes (dendritic arborization, synaptogenesis, and axonal sprouting), enhanced brain activity, angiogenesis, neurogenesis, and the release of growth factors (brain-derived neurotrophic factor (BDNF), growth-associated protein 43 (GAP43)). Importantly, the upregulation of the aforementioned processes and growth factors play a significant role in facilitating motor and cognitive recovery following ischemic stroke. As discussed above, EE is multi-faceted, incorporating a number of behavioral experiences. The mechanisms upregulated in response to EE alone, or in combination with other components of ER paradigm (exercise, task-specific training), are discussed in relation to their role in promoting recovery following stroke (Table 1).

### Structural Changes (Dendritic Arborization, Synaptogenesis, Axonal Sprouting, White Matter, Lesion Volume)

While some have demonstrated reduced lesion volume following EE (Buchhold et al., 2007; Zhang et al., 2017), the vast majority of studies do not show a difference in the size of the infarct in standard housed animals compared to EE (Johansson and Ohlsson, 1996; Biernaskie and Corbett, 2001; Risedal et al., 2002; Hirata et al., 2011; Clarke et al., 2014; Madinier et al., 2014). In fact, if EE is introduced within the first few days after stroke it can increase infarct volume and cell loss (Risedal et al., 1999; Farrell et al., 2001). These findings indicate that the beneficial effects of EE for stroke recovery go beyond simple neuroprotection.

A prevailing view of how stroke rehabilitation reduces neurological impairments is by enhancing use-dependent activation of intact tissue adjacent to the infarct and contralesional cortical regions, thereby shaping neural reorganization (Nudo et al., 1996a,b; Dijkhuizen et al., 2001; Binkofski and Seitz, 2004). Experience-induced plasticity following stroke results in remodeling of dendrites in perilesional tissue, and possibly protects vulnerable neurons from further damage (Johansson and Belichenko, 2002; Brown et al., 2008). In healthy rats, EE alone also increases dendritic spines in all cortical layers (Johansson and Belichenko, 2002), while social isolation has been reported to have the opposite effect (Bryan and Riesen, 1989). In hypertensive rats, EE following MCAo increases dendritic spines in pyramidal neurons in layers II/III compared to standard housing conditions (Johansson and Belichenko, 2002). Further, pairing a task-specific reaching paradigm with EE 15 days after MCAo results in increased basilar dendritic growth in layer V pyramidal neurons within the uninjured motor cortex, and corresponding improved functional recovery (Biernaskie and Corbett, 2001). Similarly, EE promotes synaptogenesis in perilesional and contralesional cortex and enhances use-dependent activity in perilesional cortex compared to standard housing (Jones et al., 1999; Hirata et al., 2011; Clarke et al., 2014). Following MCAo the change in synaptic density and structure following 2 weeks of EE has also been associated with improved functional recovery on a spatial memory task (Xu et al., 2009). Further, both exercise and EE enhance axonal sprouting and reduce white matter damage (Papadopoulos et al., 2009; Li et al., 2015; Hase et al., 2017, 2018). Running wheel exercise, often included in EE paradigms and associated with improved functional recovery, enhances axonal remodeling following focal cortical stroke (Li et al., 2015). In models of chronic hypoperfusion, glial damage in white matter, and neuroinflammation, is also attenuated in mice exposed to EE (Hase et al., 2017, 2018). Similarly, chronic cerebral hypoperfusion and oxidative stress in the hippocampus are prevented following 12 weeks of EE in rats, likely due to heightened antioxidant enzyme activity (Cechetti et al., 2012).

### Vasculature

The cerebrovasculature plays a potentially important role in promoting post-stroke recovery (Ergul et al., 2012). Following stroke, angiogenesis is upregulated in order to increase blood flow to damaged tissue and thereby engage endogenous recovery mechanisms such as synaptogenesis, synaptic plasticity and neurogenesis. Similar to the proangiogenic effects of exercise alone (Hu et al., 2010; Matsuda et al., 2011; Zheng et al., 2011; Yang et al., 2012; Ma et al., 2013), EE delivered in the recovery period following ischemic stroke can stimulate angiogenesis throughout the brain and perilesional tissue through vascular endothelial growth factor (VEGF), fibroblast growth factor-2 (FGF-2), and astrocytic high-mobility group box-1/interleukin-6 (HMGB1/IL-6) signaling (Seo et al., 2013; Yu et al., 2014; Chen et al., 2017; Zhang et al., 2017). Importantly, these changes in the cerebrovasculature occur in parallel with varying degrees of functional recovery post-stroke such as grip strength, motor coordination and function (Seo et al., 2013; Yu et al., 2014), decreased depression and anxiety (Chen et al., 2017), and enhanced learning and memory (Yu et al., 2014). Additionally, EE also attenuates blood brain barrier leakage following focal cerebral ischemia and in models of vascular cognitive impairment (Hase et al., 2017; Zhang et al., 2017).

### Neurogenesis

Migration of new immature neurons to the site of stroke damage has been shown to occur following ischemic cell death, and in close association with newly formed vasculature (Ohab et al., 2006). Significant literature has demonstrated the benefit of EE on neurogenesis concurrent with enhanced spatial learning and memory (van Praag et al., 2000; Simpson and Kelly, 2011; Leger et al., 2015). Likewise, enhanced neurogenesis is recognized to be upregulated following EE in different models of stroke (Komitova et al., 2005b, 2006; Buchhold et al., 2007; Wurm et al., 2007; Venna et al., 2014). For example, after MCAo in rats, both early (24 h post-stroke) and late (7 days post-stroke) administration of EE for 5 weeks results in significantly more newly born cells in both ipsi- and contra-lateral cortical regions than standard housing (Komitova et al., 2006). This increase in neurogenesis is often accompanied by improved cognitive and sensorimotor function (Komitova et al., 2005a; Wurm et al., 2007). Furthermore, the exercise component of EE may be largely responsible for these neurogenic effects (Grégoire et al., 2014), which is confounded by findings that exercise also results in upregulation of many neuroplasticity-promoting factors such as BDNF (Bechara and Kelly, 2013). This suggests that although neurogenesis and post-stroke recovery may occur in tandem, this may be coincidental, with recovery being more directly related to the upregulation of a variety of growth-promoting factors such as BDNF and GAP43 (Rossi et al., 2006; Ploughman et al., 2009; Clarkson et al., 2011; Mizutani et al., 2011; Cook et al., 2017).

### Growth Promoting and Inhibitory Factors

Both the early phase following stroke and initiation of EE are associated with an increase in growth promoting factors (glial-derived synaptogenic thrombospondin 1 and 2, GAP43, MARKS, CAP23, BDNF, etc.) that have varying effects on the aforementioned changes in neuronal structure (Murphy and Corbett, 2009). Thus, the timing of when rehabilitation is delivered is important, with the goal to actively engage in this early time period post-stroke (Corbett et al., 2015). BDNF has a major role in spontaneous and rehabilitation-induced recovery following stroke (Ploughman et al., 2009; Clarkson et al., 2011; Cook et al., 2017). For example, administration of BDNF intravenously or via a hydrogel significantly improves tissue repair and motor recovery in two different rodent models of stroke (Schäbitz et al., 2004; Cook et al., 2017). While EE increases BDNF in some studies of ischemic brain injury (Gobbo and O’Mara, 2004; Venna et al., 2014), others have reported negative findings (Risedal et al., 2002; Hirata et al., 2011). However, it is important to note that rehabilitation and exercise intensity are significant determinants as to whether rehabilitation is accompanied by increases in BDNF and whether significant functional recovery occurs (Ploughman et al., 2007; MacLellan et al., 2011a). Likewise, in the perilesional cortex of rats with cortical injury, running wheel exercise has been associated with increased GAP43, as well as its phosphorylated form (pSer41-GAP43), a key protein involved in neuronal plasticity (Mizutani et al., 2011). Other neurotrophic factors such as insulin-like growth factor-1 (IGF-1), FGF-2, nerve growth factor (NGF) and neurotrophin-3 (NT-3) are also increased by varying amounts of EE (Hu et al., 2013; Seo et al., 2013; Yu et al., 2016).

A critical window for stroke recovery has been linked to post-stroke upregulation of growth promoting factors (described above), with closing of this window related to the upregulation of growth inhibiting genes, such as NOGO and chondroitin sulfate proteoglycans (CSPGs; Murphy and Corbett, 2009). In order for recovery to occur beyond this finite period, interventions should attempt to promote a more permissive environment for neuroplasticity and recovery. For example, administering chondroitinase ABC, which degrades inhibitory CSPGs in the extracellular matrix, or blocking neurite inhibitory protein Nogo-A, enhances sensorimotor recovery following focal stroke due to new axonal connections and increased dendritic arborization in contralesional cortex (Papadopoulos et al., 2002, 2006; Soleman et al., 2012). Similarly, providing EE for 9 weeks after photothrombotic stroke results in a reduction of aggrecan-containing perineuronal nets surrounding parvalbumin containing GABAergic neurons in the peri-infarct area (Madinier et al., 2014). Additionally exercise results in a downregulation of Nogo-A signaling in perilesional tissue, promoting axonal remodeling (Li et al., 2015).

Establishing which EE-induced mechanisms are critical for stroke recovery is difficult to investigate experimentally, with the vast majority of studies being correlative in nature. A substantial body of preclinical work has focused on the potential role of neurogenesis, yet the precise role of neurogenesis or the degree to which it occurs in adult humans has recently been questioned (Sorrells et al., 2018). Nonetheless, the aforementioned mechanisms and processes discussed above likely have a collective role in promoting recovery following stroke rather than any single one. Indeed, the post-stroke time course of these neuroplasticity processes strongly relate to the functional recovery observed across different domains (cognitive, sensorimotor, etc.).

## Benefits of Environmental Enrichment on Functional Recovery in Animals

### Sensitive Periods Following Stroke: The Importance of Maximizing Therapy Dose in the Early Post-stroke Phase

Corresponding with the aforementioned changes in growth factors, recovery of post-stroke motor impairment is thought to plateau within the first 4–5 weeks in rodents (Biernaskie et al., 2004; Murphy and Corbett, 2009) and the first 3–4 months in humans (Jørgensen et al., 1995; Kwakkel et al., 2006; Langhorne et al., 2011), with a large degree of improvement during this time being attributable to spontaneous recovery in both species (Prabhakaran et al., 2008; Krakauer et al., 2012; Winters et al., 2015; Jeffers et al., 2018a,b). Although recovery can still occur outside of this period, these changes may be mediated by compensatory strategies, rather than restitution of neurological impairments (Zeiler and Krakauer, 2013). This highlights the need for preclinical work to consider more sensitive measures of sensorimotor recovery, such as kinematics (Corbett et al., 2017). Furthermore, although some degree of recovery may occur at any time following stroke, the rate of change becomes more limited as time post-stroke increases (Lohse et al., 2016). Evidence from both preclinical and clinical studies suggest that rehabilitation therapies should be maximized in the early weeks and months following stroke, with caution being taken to not intervene too early (i.e., <3 days), when intensive therapy may have contradictory, or even detrimental effects (Humm et al., 1998; Risedal et al., 1999; Farrell et al., 2001; Dromerick et al., 2009; Lang et al., 2015; Langhorne et al., 2017).

Despite some experiments not finding a relationship between therapy dose and recovery (Winstein et al., 2016), overall meta-analysis across clinical trials have indicated that increased therapy dose augments recovery across a range of post-stroke impairments, using a variety of intervention strategies and outcome measures (Lohse et al., 2014; Schneider et al., 2016). Additionally, the benefits of post-stroke task-specific training have been shown to be transferrable to non-trained tasks (Schaefer et al., 2013). As rehabilitation resources are often limited, alternative methods for increasing therapy dose are highly desirable. EE may provide one such adjunctive intervention for increasing non-specific therapy dose, as this treatment paradigm provides a stimulating environment that enhances stroke recovery in rodents across a variety of impairment domains without requiring provision of specific training (Ohlsson and Johansson, 1995; Risedal et al., 2002; Livingston-Thomas et al., 2016). Furthermore, this stimulating environment has a potentiating effect on task-specific therapy, resulting in recovery beyond what would have occurred with either EE, or task-specific therapy alone (Jeffers and Corbett, 2018).

### Efficacy of Environmental Enrichment in Non-motor and Motor Recovery Domains

As previously mentioned, early work with EE focused on how stimulating early life experience promotes enhanced cognitive development (Hebb, 1947). Later, cortical injury models in rodents were used to probe the various functions and network connectivity of the brain, while investigating how early-life EE could ameliorate impairments in learning and memory associated with these injuries (Kolb and Elliott, 1987; Kolb and Gibb, 1991). EE’s efficacy in improving cognitive function in these studies led to utilization of this treatment for adult focal ischemia in rodents, with a continued focus on cognitive performance. Following stroke, EE has been shown to significantly enhance spatial learning of the Morris Water Maze (Risedal et al., 1999; Dahlqvist et al., 2004; Rönnbäck et al., 2005; Sonninen et al., 2006) and spatial memory in Radial Arm Maze tasks (Buchhold et al., 2007). These benefits appear to be robust across injury types, as similar benefits of EE have been observed in Morris Water Maze acquisition (Puurunen et al., 1997) and switching between relevant reward-cues in the Win/Shift-Win/Stay version of the T-maze task (Farrell et al., 2001) in models of global ischemia. EE also alleviates depression-like behaviors in mice (Jha et al., 2011), which is an important consideration, as depression in humans after stroke is common (Arwert et al., 2018). Overall, these studies (see Table 2) demonstrate the robust cognitive benefits of EE, and the potential for this treatment to be applied to other domains of impairment in preclinical models of stroke.

**Table 2:** Benefits of EE on functional recovery in animals following stroke.

Benefits	Task	References
↑ Spatial learning	Morris Water Maze	Puurunen et al. (1997), Risedal et al. (1999), Dahlqvist et al. (2004), Rönnbäck et al. (2005) and Sonninen et al. (2006)
↑ Spatial memory	Radial Arm Maze	Buchhold et al. (2007)
↑ Working memory	T-maze	Farrell et al. (2001)
↓ Depression-like behaviors	Tail suspension test, open-field and sucrose preference test	Jha et al. (2011)
↑ Motor recovery	Rotarod	Ohlsson and Johansson (1995), Johansson (1996), Johansson and Ohlsson (1996), Nygren and Wieloch (2005), Nygren et al. (2006) and Buchhold et al. (2007)
	Ladder crossing	Biernaskie et al. (2004), Windle et al. (2007) and Wurm et al. (2007)
	Limb placement	Puurunen et al. (2001)
	Adhesive strip removal	Kuptsova et al. (2015)
	Montoya staircase	Biernaskie and Corbett (2001) and Jeffers et al. (2014)
	Single pellet reaching	Jeffers and Corbett (2018)

*Up and down arrows indicate an increase or decrease in the corresponding factor in response to EE, respectively*.

The preclinical stroke field has primarily used EE to promote motor recovery and study its underlying neuroplastic mechanisms. Many studies have demonstrated benefits of EE on post-stroke recovery of a variety of sensorimotor tasks (see Table 2), including: rotarod (Ohlsson and Johansson, 1995; Johansson and Ohlsson, 1996; Johansson, 1996; Nygren and Wieloch, 2005; Nygren et al., 2006; Buchhold et al., 2007), ladder crossing (Biernaskie et al., 2004; Windle et al., 2007; Wurm et al., 2007), limb placement (Puurunen et al., 2001), and adhesive strip removal (Kuptsova et al., 2015). While some studies have shown neutral, or slightly negative effects of EE on similar sensorimotor tasks (Hicks et al., 2008), meta-analysis of these results indicates that EE has a significant benefit on general sensorimotor function (Janssen et al., 2010). Furthermore, these benefits also extend to models of intracerebral hemorrhage (Auriat and Colbourne, 2008), which receives relatively little attention compared to focal ischemia in the preclinical literature.

One caveat to this positive outlook on EE for enhancing motor recovery is that tasks of fine motor dexterity, such as pellet retrieval, do not demonstrate the same benefits as less-skilled motor outcomes (Grabowski et al., 1993; Ohlsson and Johansson, 1995; Auriat and Colbourne, 2008; Kuptsova et al., 2015). As such, EE may not substitute for task-specific (e.g., upper limb) therapy; however, it could potentially serve as an adjunct to conventional care that would enable greater recovery than possible with task-specific training alone (Livingston-Thomas et al., 2016). This adjunctive approach to EE and task-specific training is supported by evidence that such combination therapies augment recovery of fine-motor skills that normally do not benefit from EE alone, in both models of focal ischemia (Biernaskie and Corbett, 2001) and intracerebral hemorrhage (MacLellan et al., 2011b; Caliaperumal and Colbourne, 2014). Additional combinations of EE with various pharmacological agents has also yielded promising synergistic results; however, this work is still in its infancy (Corbett et al., 2014; Mering and Jolkkonen, 2015; Malá and Rasmussen, 2017). Our previous work has demonstrated that the combination of EE, task-specific reaching and growth factor administration accelerates the rate of recovery of fine motor dexterity (Jeffers et al., 2014). Studies such as these further emphasize that the naturalistic behaviors and heightened activity encouraged by EE has the potential to produce a powerful synergistic interaction to promote recovery of even very specific skilled functions post-stroke (Zeiler and Krakauer, 2013; Corbett et al., 2015).

### Generalization of the Benefits of Environmental Enrichment

An important consideration in attempting to translate a potential preclinical stroke treatment, such as EE, to human clinical practice is the robustness of the benefits observed in the preclinical environment. Stroke is a heterogeneous disorder, affecting both sexes at all points throughout the lifespan, causing damage in diverse brain regions and an array of functional impairments (Ramsey et al., 2017). In contrast, preclinical rodent studies of stroke typically utilize young adult, male rats, with cortical lesions that do not represent those most commonly observed in clinical studies (Edwardson et al., 2017). These factors hamper the translation of preclinical stroke treatments to clinical practice, and have led to concerted international efforts to better align preclinical and clinical experimental methodologies in stroke (Bernhardt et al., 2017a; Corbett et al., 2017). As a general principle, before considering translation to the clinic, a potential preclinical therapy should demonstrate robust benefits across a range of experimental conditions.

Undoubtedly EE has been studied under an array of conditions and preclinical demographics (Simpson and Kelly, 2011). In addition to the diverse benefits outlined above, EE has also been shown to exhibit significant effects throughout the lifespan, from neonatal (Kolb and Gibb, 1991; Rojas et al., 2013) to aged animals (Buchhold et al., 2007). However, with aging, animals may need to be subjected to more intense stimulation than younger animals in order to obtain the same benefits of EE (Bennett et al., 2006). The literature regarding sex-differences in the efficacy of EE is much less clear. Studies have shown greater benefits of EE for females (Pereira et al., 2008), males (Langdon et al., 2014), or similar effects between sexes (Frick et al., 2003; Saucier et al., 2010; Schuch et al., 2016). As only ~17% of EE studies have included both male and female animals, and of this subset only a minority of studies has been concerned with the effects of stroke, or stroke recovery, it is unlikely that enough data currently exists in the literature to definitively answer the conditions under which sex-specific effects of EE may occur (Simpson and Kelly, 2011). As previously outlined, EE has shown beneficial effects for both cognitive and motor recovery using a variety of models of neurological damage including: global ischemia (Farrell et al., 2001), neonatal hypoxia-ischemia (Pereira et al., 2007; Rojas et al., 2013), intracerebral hemorrhage (Auriat and Colbourne, 2008), and cortical injury in a variety of regions using different lesion induction methods (Kolb and Gibb, 1991; Johansson, 2004; Buchhold et al., 2007; Windle et al., 2007; Jeffers et al., 2014; Kuptsova et al., 2015). Another important consideration is whether the beneficial effects of EE are lasting, since the vast majority of preclinical EE studies maintain enrichment until the time of sacrifice. One study provided ER for 9 weeks, at which time animals post-stroke recovery had plateaued. Thereafter, animals were given two cycles (“tune-ups”) of 5 weeks of no treatment followed by 2 weeks of additional ER. However, these tune-ups provided no additional benefits to recovery. Re-testing throughout this period revealed that the initial functional gains from the first 9-week exposure to ER were maintained, suggesting the benefits of ER are long lasting (Clarke et al., 2009). The demonstrated efficacy of EE across a wide variety of stroke models and conditions, together with the overall positive effects on stroke recovery in meta-analysis, suggests that EE may be an ideal intervention for clinical trial assessment (Janssen et al., 2010).

## Environmental Enrichment as an Adjunctive Therapeutic in Humans

### Current State of Post-stroke Activity Levels

Despite the above-mentioned literature highlighting the importance of experience to shape behavior and recovery, people with stroke who are inpatients in hospital have limited exposure to a range of experiences, activities and therapy opportunities. A large body of evidence has demonstrated that stroke patients in hospital (up to 3 months post-stroke) consistently exhibit an activity profile of “inactive and alone”. In 2004, Bernhardt et al. reported that stroke patients spend 50% of their time resting in bed, 88.5% in their bedroom and 60% of time alone (Bernhardt et al., 2004) and little has changed in the ensuing years. Patients remain inactive, alone and in their bed/bedroom for large proportions of the day (Table 3, Fini et al., 2017). While evidence is limited, it also appears that stroke patients demonstrate low levels of social and cognitive activity: in acute care, social activity represented ~29.3% of time observed, while cognitive activity represented ~44.7% of time (Rosbergen et al., 2016) and in subacute rehabilitation, social activity occurred in 32% of observations and cognitive activity in only 4% of observations (Janssen et al., 2014).

**Table 3:** % of observed time in bed, in bedroom and alone.

Study	Location	% Observations in bed	% Observations in bedroom	% Observations alone
Bernhardt et al. (2004)	Acute	50	88.5	60
Askim et al. (2012)	Acute and subacute	30.3	−	−
Åstrand et al. (2016)	Acute group	33	82	54
	Subacute group	21	53	52
English et al. (2014)	Subacute	0	55	47
Hokstad et al. (2015)	Acute and subacute	44	74	56
Janssen et al. (2014)	Acute and subacute		Inactive and alone 40
King et al. (2011)	Subacute	52	76	47
Prakash et al. (2016)	Acute and subacute	52	15	78
Rosbergen et al. (2017)	Acute	68	94.5	58.9
Skarin et al. (2013)	Subacute	38	−	52
van de Port et al. (2012)	Acute and subacute	62	87	61
West and Bernhardt (2013)	Acute and subacute	60	76.1	51.9

These low activity levels of stroke patients raise concerns regarding the rehabilitation environment and demonstrates that little patient-initiated therapeutic activity (i.e., without a therapist) occurs during acute and subacute stroke rehabilitation. Synthesizing perspectives and preferences of stroke patients in acute and subacute inpatient rehabilitation shows that patients highly value physical activity and believe that physical activity levels are highly related to enhanced recovery (Luker et al., 2017). Stroke survivors indicate that they want to practice meaningful activities and have more opportunities to engage in recreational activities (Luker et al., 2017). Indeed, a recent review showed that boredom was a very common experience during inpatient rehabilitation for patients with acquired brain injuries (Kenah et al., 2017). Patients highlight that communal areas and outdoor spaces, which provide opportunities for engagement in activities, reduce boredom (Kenah et al., 2017). Importantly, patients recognize that current inpatient rehabilitation is not meeting their activity needs and remain insufficiently engaging.

Animal studies of ER have provided opportunities for very intensive therapy, whereas human stroke patients are typically limited in this regard. From observational studies, direct therapist time focused on active upper limb therapy has been found to be <5 min per day in the acute setting and <17 min per day in the subacute setting (Hayward and Brauer, 2015), and consistent with ~32 repetitions (Lang et al., 2009). With regards to lower limb activities, Fini et al. (2017) reported across acute and subacute settings, 9.2% of therapy time was directed to standing and walking. Mean time spent walking was 31 min per day in subacute rehabilitation, with likely even less time spent on walking in acute stroke units as patients are more dependent early after stroke.

As outlined above, the present clinical setting contrasts dramatically with preclinical EE and ER where animals are exposed to a high level of social interaction, cognitive stimulation, opportunities for physical activity and intensive rehabilitation to achieve sensorimotor stimulation (Biernaskie et al., 2004). Therefore, optimization of how stroke patients spend their day in acute or subacute inpatient rehabilitation after stroke may be an avenue for improving stroke outcome by emulating preclinical EE in patient care.

### Optimizing the Post-stroke Environment

It is essential to explore alternative opportunities to promote greater social, cognitive, and physical activity post-stroke. EE and ER may be a critical aspect that has been long overlooked in rehabilitation units. Similar to animal models, a natural environment for a human is quite enriched; however, hospital environments have been generally considered to be impoverished. An EE is a non-direct therapy approach that can help to equip stroke survivors with the skills to drive their own activity levels and recovery (Barker and Brauer, 2005). Creating an EE that stimulates activity beyond direct therapy time is an important line to explore in the clinical setting and could address the needs of therapists and stroke survivors. While translation is in its infancy, there are global efforts to learn from animal models of enrichment and translate the EE and ER approach to human stroke rehabilitation settings. This line of research will be discussed in order of stroke progression (i.e., acute to subacute), but will not include enrichment strategies that target a specific activity domain alone such as physical activity through group therapy (English et al., 2015), personalized out of therapy protocols (Harris et al., 2009); or social activity using groups (Higgins et al., 2005).

#### Translation to Acute Stroke Unit

The acute stroke unit is a unique rehabilitation environment, as the majority of stroke patients are more dependent and require frequent assistance from staff to undertake activities. The EE adaptation tested by Rosbergen et al. (2017) in the acute stroke unit included access to communal areas with a variety of equipment to enhance activities away from the bedside including iPads, books, puzzles, newspapers, games, music and magazines available 24 h a day. Daily group sessions (1-h duration) were provided with a focus on different aspects of stroke recovery such as stroke education, emotional support, communication and upper limb, balance, mobilization activities. An opportunity for communal breakfast and lunch was included to stimulate frequency of mobilization and social interaction, as well as encourage sitting upright for mealtimes (Rosbergen et al., 2016). In addition to environmental changes, stroke patients and families received information that explained the importance of activity after stroke, outlined organizational structure of the unit and how stroke patients and families could contribute to encourage activity out of therapy hours (Rosbergen et al., 2016). Under this protocol, the EE group (*n* = 30) spent a significantly higher proportion of their day engaged in “any” activity (71% vs. 58%) compared to the usual care group (*n* = 30) and were significantly more active in physical (33% vs. 22%), social (40% vs. 29%) and cognitive domains (59% vs. 45%). Furthermore, the enriched group experienced significantly fewer adverse events (e.g., falls), with no differences found in serious adverse events (e.g., death). The increased activity levels remained evident in the acute stroke unit environment 6-months post-implementation of the EE paradigm.

#### Translation to Inpatient Rehabilitation

Janssen et al. (2014) focused on access to communal and personal enrichment spaces with the view to increase activity that was driven by the environment. Patients were recruited during the first 4 weeks post-stroke and communal enrichment strategies included computers with internet connection, reading material, jigsaw puzzles, board games and tablets. Strategies targeting personal enrichment were also used and included access to music, audio books, books, puzzles and board games; family members were encouraged to bring in hobbies and activities that patients enjoyed pre-stroke; staff were advised to encourage stroke patients to access communal areas or use personal enrichment resources when patients were observed inactive. Per this 2-week protocol, Janssen et al. (2014) demonstrated that stroke survivors engaged in an EE were: (a) 1.2 times more likely to do “any activity” compared to individuals with stroke in the control group with no EE (activity change from timepoint 1 to timepoint 2 (ΔT1-T2): 13% EE vs. 2% control observations); (b) 1.1 times more physical (ΔT1-T2: 8% EE vs. 5% control); (c) 1.2 times more social (ΔT1-T2: 3% EE vs. −5% control); and (d) 1.7 times more cognitively active (ΔT1-T2: 7% EE vs. 1% control). This pilot study was small (*n* = 15 intervention group) but was a critical piece of translation work showing how the field is beginning to approach the post-stroke environment.

An alternative approach to enrichment was explored by Khan et al. (2016) in a larger sample using a randomized controlled trial (*n* = 103, 51% stroke survivors). Individual and communal EE was offered, including an activity stimulating area, the “activity arcade.” In contrast to Janssen, where access to activities was available throughout the entire day, in Khan et al. (2016), access to the activity arcade was for 2-h per day only. Activities provided in the arcade were consistent with Janssen et al. (2014) including computers with internet access; workstations with gaming technology; books; music; life-size mirrors for visuo-perceptual deficits; as well as novel training tasks including simulated shopping corner with groceries, electronic payment machines, and bank teller machines; wood workshop, and other activities. This multifaceted approach is more comparable to preclinical EE, where rodents are exposed to a variety of activities in enrichment chambers (Hannan, 2014). Findings (for stroke patients only) demonstrated significant improvements in depression (Depression Anxiety Stress Scale, DASS mean difference from baseline −24.1 (95%CI −40.1, −7.2) and general function (Functional Independence Measure motor, FIM-motor mean difference from baseline 6.7 (95%CI 0.2, 13.1) at discharge compared to the control group, who received standard therapy on the ward at the same time as enrichment patients. However, no differences in Cognition (Montreal Cognitive Assessment and FIM-cognition) and overall health (EQ-5D) were found between groups and improvements were not maintained within patients at 3-months follow-up. As observation of activity levels was not an outcome measure, the impact of enrichment on activity levels remains unknown.

Collectively the studies completed to date demonstrate important outcomes in activity and function, as well as the ability to embed adjunctive indirect therapy through enrichment of the environment within acute and subacute rehabilitation settings.

### Contrasts Between Preclinical and Clinical Enriched Environments

To date, it is clear that the approaches used in preclinical and clinical stroke rehabilitation settings have differed. Key distinctions between animal and human stroke studies are presented in Table 4. A significant barrier to clinical implementation is configuration of the EE environment. In animal studies cages are not difficult to standardize, it is easy to increase the novelty of objects and tasks while allowing unlimited access to all areas of the cage. In human stroke rehabilitation it is much more difficult to standardize EE conditions across sites, since stroke rehabilitation units vary, some patients have limited access due to impairment levels, length of stay can vary, and due to cost restrictions, the EE cannot be physically rearranged very easily. Although no sex-specific differences in EE have been identified with regards to stroke rehabilitation, a limitation in preclinical work to date is that most studies have utilized young male rodents. While clinical EE has attempted to mirror the physical, social and cognitive focus of preclinical EE, the opportunity for more strenuous exercise, similar to rodent running wheels, is lacking. Further, few clinical studies to date have attempted to include more task-specific rehabilitation into their EE paradigm similar to ER, which preclinical work has shown to be even more advantageous than EE alone (Jeffers and Corbett, 2018). Nonetheless, taking these differences into account, there are considerable research opportunities to better align preclinical and clinical EE and ER research.

**Table 4:** Differences between preclinical and clinical housing conditions, delivered care and therapy routines.

Housing conditions	
**Preclinical EE**	**Clinical EE**
Animal cages can be built to have standardized physical environments	Stroke and rehabilitation units physical build varies widely from hospital to hospital
Easy to change housing environment	Difficult to change housing environment (e.g., built floor plan, walls and communal space locations)
Animals unlimited access to all areas	Patient with contact precautions and higher stroke severity (e.g., unable to mobilize independently) have limited access
Controlled number of animals with uniform stroke severity in the environment	Controlled number of patients, but large number of staff, visitors, and non-stroke patients also interacting in environment
Length of stay is based on biology of recovery	Length of stay is pragmatic and limited by funding
**Species, care and therapy**	
Predominantly young, male rodents	Stroke patients are largely older, mixed sex populations
Controlled daily routine	Daily routine frequently interrupted (e.g., medical investigations, visitors, medical emergencies on acute ward)
Rodents activities are spontaneous, rather than directed by a therapist	Humans activities based on learned behaviors and influenced by therapists, carers and other medical staff
Rodents can engage in any activities as soon as they desire, at any level of intensity (not restricted by investigator)	Human activities may be restricted by care givers (e.g., number of people to assist to mobilize) and/or hospital procedures (e.g., safety measures to prevent falls)
Rodents access only the cage	Humans have access to areas beyond the unit e.g., therapy spaces, outdoor areas, hospital grounds and beyond
Rodent EE encourages more physical, social, and cognitive activity and often contains a variety of self-initiated opportunities for exercise, and in ER, includes intensive reaching practice	Human EE also encourages more physical, social, and cognitive activity, but has fewer opportunities for strenuous exercise or task-specific reaching practice

### Implementation of EE in Clinical Practice: Are We Ready?

Before wide-spread implementation of EE in a clinical setting, stronger evidence for its benefits in post-stroke patients is required. So far, no large scale clinical trials of effectiveness and cost efficacy have been undertaken (e.g., Phase III). To date, the few small to medium sized studies (*n* = 14 to *n* = 52 stroke patients) have demonstrated that activity levels can be increased (Janssen et al., 2014) and appear to remain sustained over time within units (Rosbergen et al., 2017), but not within individuals (Khan et al., 2016). However, we have limited evidence of improved stroke recovery in terms of disability (e.g., modified Rankin Scale), function (e.g., Fugl Meyer Assessment, Action Research Arm Test, walking ability) or participation (e.g., return to meaningful activities); nor evidence of biological changes (e.g., altered functional connectivity, growth factors, etc.) like that found in animal models. It is likely that enrichment is one piece of a complex rehabilitation intervention and thus, trial design is challenging.

There is considerable cause for optimism that EE can increase stroke patient activity indirectly, but potential translational roadblocks need to be addressed prior to wide-spread implementation of EE in a clinical setting. There is a need to consider how we best design an effectiveness trial (e.g., cluster trial), but to progress translation of EE to the clinical setting we need early phased studies as well. Such studies need to focus on building an understanding of how EE works, focusing on the neurobiology and individual differences. While human research cannot always probe the same biological mechanisms available to preclinical research, human studies can use data collected preclinically to guide key biomarkers of interest for the clinic (Boyd et al., 2017). This includes using functional imaging such as resting and functional MRI, EEG and MEG to understand the influence of EE on cortical and subcortical networks, as well as TMS to investigate cortical excitability and inhibitory patterns. Further, structural changes at the macrolevel can be probed, for example using diffusion weighted imaging to explore whole brain white matter fiber integrity, as well as various MRI scans to model microlesion load. Inclusion of blood (to model potential growth-promoting and inflammatory biomarkers) and genetic (to explore BDNF polymorphisms) assays could also be included to help understand who might benefit most from EE. Exploring biomarker candidates that have been identified in parallel preclinical research may also inform stratification of patients in future trials (Jeffers et al., 2018b).

A better understanding of the optimal dose of EE is required. Trials that attempt to understand the dose characteristics of EE could use novel 3 + 3 designs that progressively increase exposure across physical, social, and cognitive activities that may shape behavior. This can allow sophisticated and detailed analysis of the effect of EE on activity levels, well-being, functional outcomes and fatigue levels. As well, any models of EE must consider the impact of ER evidence in animals. We cannot assume that EE alone will be the recovery breakthrough without considering the need to substantially increase the dose of complex and challenging therapy opportunities. While human studies use behavioral mapping to profile individual patient activities, technological advancements have also enabled rodent tracking on the individual level, using methods such as video shape recognition, or RFID tagging. This alignment of preclinical and clinical research methodologies will enable parallel, and complementary, research to be conducted across species in order to determine the optimal EE environment for promoting neuroplasticity and stroke recovery.

Finally, EE requires the environment to be novel and complex. At present there are limited opportunities for stroke patients to engage in physical, social and cognitive activities within the inpatient rehabilitation environments. To enable access to meaningful activity for stroke patients there is a need to create activities that are accessible outside of therapy. Self-directed upper limb and mobility activities, including smart use of technology such as gaming, robotics and virtual reality may contribute to enhance EE translation.

## Future Directions

As discussed by the international Stroke Recovery Roundtable group, for stroke recovery research to progress forward there is a need for closer alignment of preclinical and clinical research (Bernhardt et al., 2017a,b; Boyd et al., 2017; Corbett et al., 2017). Despite a significant amount of preclinical research being conducted on the ability of EE and ER to enhance stroke recovery, questions still remain to translate this adjunctive model of therapy to the clinic. For example, while rehabilitation strategies that promote neuroplasticity are important for functional recovery following stroke it is also recognized some forms of neuroplasticity may actually be maladaptive (Jones, 2017). Training the unaffected limb on a reaching task following focal stroke actually worsens behavioral recovery in the affected limb (Allred and Jones, 2008). This maladaptive plasticity is mediated by transcallosal projections (Allred et al., 2010), and has also been linked to abnormal synaptogenesis and decreased neural activation of perilesional cortex (Allred and Jones, 2008; Kim et al., 2015). To lessen the potential for aberrant neuroplasticity when engaging in rehabilitation, such as EE, it is important to try and limit compensatory strategies using the unaffected limb. However, the way in which EE may promote or negate compensatory strategies and learned-nonuse of the stroke-affected limb has not been widely studied in preclinical and clinical studies.

To date, studies that have investigated different EE paradigms in the clinical setting have incorporated a number of cognitive and social components that have been shown to promote greater activity. While increasing any aspect of physical, cognitive, or social activity is important, preclinical EE also has motor components that provide the ability to engage in intense physical activity, more akin to exercise (running wheel, climbing, beam walking, etc.). Since preclinical work has shown that the effects of EE are multi-factorial in nature, to demonstrate clinical efficacy future clinical translation should attempt to better mirror animal EE environments. Integrating more opportunities for patient-initiated goal directed exercise into clinical EE would likely be quite valuable, tapping into both cognitive and motor domains. Indeed, evidence from animal work demonstrates that exercise and cognitively stimulating environments alone do not provide the same magnitude of benefits as when they are provided together (Langdon and Corbett, 2012).

On the other hand, preclinical experiments should attempt to mirror the clinical setting more closely. As previously mentioned, the majority of animal studies have used young male adult rodents (Simpson and Kelly, 2011) while within the clinical setting stroke patients’ characteristics vary widely in age, stroke features, comorbidities, and prior living situations. Further, most preclinical EE studies have also administered EE 24 h a day, something that is not achievable in the clinical setting. Experiments that mimic variables encountered in the human stroke population can further contribute to the translation of EE.

Lastly, future design of acute stroke and inpatient rehabilitation units should facilitate early rehabilitation and indirect therapeutic activity. Hospitals are currently moving away from co-location of multiple patients in a bedroom to single patient bedrooms to minimize risk of infection, which results in reduced social stimulation (Anåker et al., 2017). However, to facilitate brain repair and recovery processes after stroke the architectural layout needs to promote early rehabilitation and safe indirect therapeutic activity. In this modern era for clinical practice, there is a need to break down the barriers between the disciplines that can support optimal translation and work collaboratively across the translation pipeline (Bernhardt et al., 2017a,b). This means increasing communication between preclinical and clinical researchers, as well as architecture and technology experts, and health care consumers (i.e., patients and caregivers) to create optimal health environments for stroke survivors that promote activity and recovery. Co-design is a novel methodology that could be integral to unravelling the translational hurdles of EE.

Decades of preclinical research have established that EE is a robust intervention for fostering brain plasticity and recovery from various types of brain injury, including stroke. A number of important questions remain regarding the optimal delivery of EE for promoting recovery from stroke. However, aligning the preclinical and clinical approaches to these questions may greatly accelerate our ability to undertake these challenges, and to work towards implementation of EE into the clinical domain on a large scale.

## Author Contributions

MM, KH, IR, MJ and DC contributed to the conception, literature search, drafting and revising of the manuscript. Furthermore, all authors approve the publication of this content and agree to be accountable for all aspects of the work.

## Conflict of Interest Statement

The authors declare that the research was conducted in the absence of any commercial or financial relationships that could be construed as a potential conflict of interest.
